# Bracing in adult with scoliosis: experience in diagnosis and classification from a 15 year prospective study of 739 patients

**DOI:** 10.1186/s13013-016-0090-y

**Published:** 2016-10-14

**Authors:** Jean Claude de Mauroy, Cyril Lecante, Frédéric Barral, Sophie Pourret

**Affiliations:** 1 Department of Orthopaedic Medecine, Clinique du Parc, 155 Boulevard Stalingrad, Lyon, 69006 France; 2 Orten, 125 Rue Bataille, Lyon, 69008 France

## Abstract

**Background:**

Despite the frequency of adult scoliosis, very few publications concern the conservative orthopaedic treatments. The indications have not been defined to date. The experience of a department specialized in rigid bracing allows us to consolidate and clarify diagnosis and indications as well.

**Methods:**

Individual observational prospective cohort study from a database started in 1998, with selection of all 739 adult scoliosis patients for which conservative orthopaedic treatment has been proposed to, even in case of drop-out. Scoliosis treated during adolescence and monitored in adulthood are included if a new brace is prescribed.

A first descriptive study of the main parameters was performed: gender, age, Cobb angle.

A tentative classification according to aetiology, age and angulation is proposed.

**Results:**

1. Descriptive Data:
  The Ratio Female/Male is 88 %, the mean age: 56.97 ± 15.82, the mean Cobb angle: 35.58 ± 17.35. The rate of non-adherent patients not wearing the brace is 17 % (but the plaster cast before bracing was routinely proposed at the time).
2. All patients can be grouped into five diagnoses, all statistically different, according to the age and the initial Cobb angle:
  Rotatory dislocation: 361 cases, age: 59.73 ± 13.52 (*p* = 0.05), (Cobb 39.08 ± 16.59 (*p* = 0.02)*
  Instability and disc disease: 150 cases, age: 46.03 ± 15.49 (*p* = 0.00)*, Cobb: 25.29 ± 12.29 (*p* = 0.00)*
  Camptocormia: 68 cases, age: 69.78 ± 12.19 (*p* = 0.00)*), Cobb: 38.09 ± 14.23 (*p* = 0.25)
  Kyphosis TL or T: 62 cases, age: 60.73 ± 15.51 (*p* = 0.07), Cobb: 43.34 ± (21.48 (*p* = 0.00)*
  Disabling pain: 33 cases, age: 48.36 ± 13.73 (*p* = 0.02)*, Cobb: 36.45 ± 25.21 (*p* = 0.78)

Treatment after surgery and in the context of a lumbar stenosis and spondylolisthesis are independent groups.

Despite the wide variety of etiologies, nearly 2/3 of patients have a discal pathology like rotatory dislocation and disc instability. For these patients a short brace can be used. Other patients usually have high kyphotic pathology as Kyphosis or camptocormia. They need a long brace.

**Conclusions:**

The wide variety of adult scoliosis makes any objective classification difficult. This first approach is intended to specify the best indications of bracing in adulthood.

1. The female ratio is slightly higher than that of the adolescent.
2. The dropout rate is high and justify improvements with adaptation of bracing to adults.
3. All proposed etiological groups are statistically significantly different.

**Electronic supplementary material:**

The online version of this article (doi:10.1186/s13013-016-0090-y) contains supplementary material, which is available to authorized users.

## Background

The evolution of scoliosis in adulthood is most often pejorative [1]. Although scoliosis in adults is 10 % of the population aged 65, conservative non-surgical orthopaedic treatment is the subject of few publications. Many reasons may explain this lack of publications.

The progression at adulthood is less linear and much more chaotic than during adolescence. As growth is the main factor of progression for AIS; in adulthood, the anatomical aetiologies are much more varied: disc, bone with osteoporosis, muscle, and postural system for the camptocormia which is characterized by forward flexion of the spine when standing or walking and disappears when lying down. It is related to atrophy of the deep muscles of extra-pyramidal origin.

The aims of the treatment are more blurred: pain, cosmetics, postural imbalance, Radiological curve progression and orthotic solutions are more limited for long braces.

Treatment time is much longer than during adolescence and there is hope placed in surgical rapid solutions.

In all published series, the diagnosis is poorly specified [2]. The most significant result seems to involve pain [3]. In some cases, bracing allows to avoid or postpone surgery [4].

It seemed interesting to publish a long-term prospective study of the solutions used in Lyon for over 50 years and attempt a classification of the main indications.

## Methods

With approval of the French CNIL (n°1880517), we retrospectively reviewed the prospective database that started in 1998.

The only inclusion criteria was the indication of a rigid brace, usually at the request of the General Practitioner. The study parameters were: age after Risser 5, Cobb angle and diagnosis. All patients are consecutive. The initial diagnosis included 18 categories that were secondarily regrouped into 8 categories (Table 1).

**Table 1:** Distribution of diagnostics. The 8 most frequent diagnoses with their percentage

1	After surgery (*n* = 86) = 11 %
2	Rotatory Dislocation (*n* = 361) = 48 %
3	Lumbar instability (*n* = 150) = 19 %
4	Disabling pain (*n* = 33) = 5 %
5	Spinal Stenosis (*n* = 5)
6	Camptocormia (*n* = 68) = 9 %
7	Thoraco-lumbar kyphosis (*n* = 62) = 8 %
8	Spondylolisthesis (*n* = 14)

Some diagnostics were grouped as the mean age and average Cobb angle showed no significant difference. For instance, discopathy, lumbar instability, dysfunction and herniated disc. Disabling pain includes: sciatica, neuropathic pain, rheumatic rigidity and disability (Additional file 1).

This is an exhaustive presentation. For example, some young patients have severe pain after Risser 5 or with Cobb angles between 10° and 30°. In fact, we don’t treat scoliosis but discal pain.

Statistics were made using the SPSS 20 pack with a Confidence interval of 95 %.

### Description of the brace system and treatment protocol

**Fig. 1:**
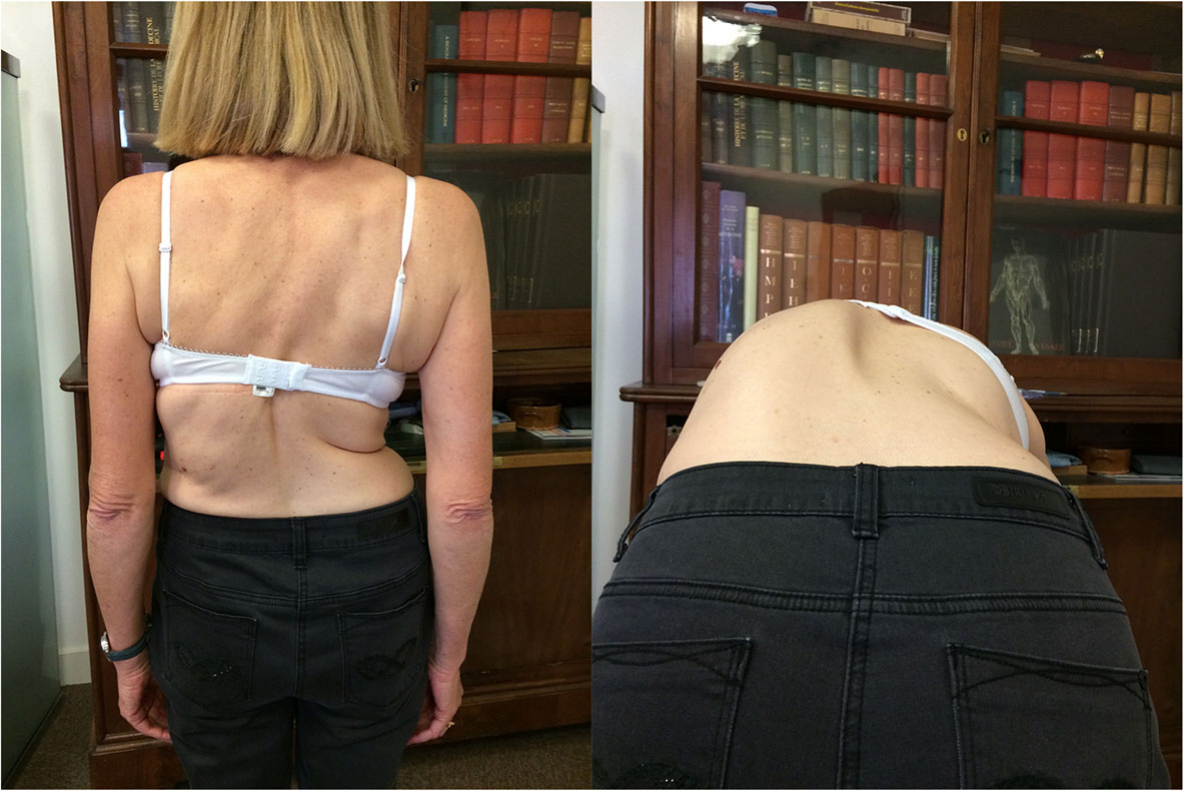
Patient with 70° scoliosis. Thoraco-lumbar scoliosis T10-L3 70°, with rotatory dislocation L3-L4. Treatment was started 12 years ago, the angulation remains stable, the brace is worn in case of pain and after sport activities

**Fig. 2:**
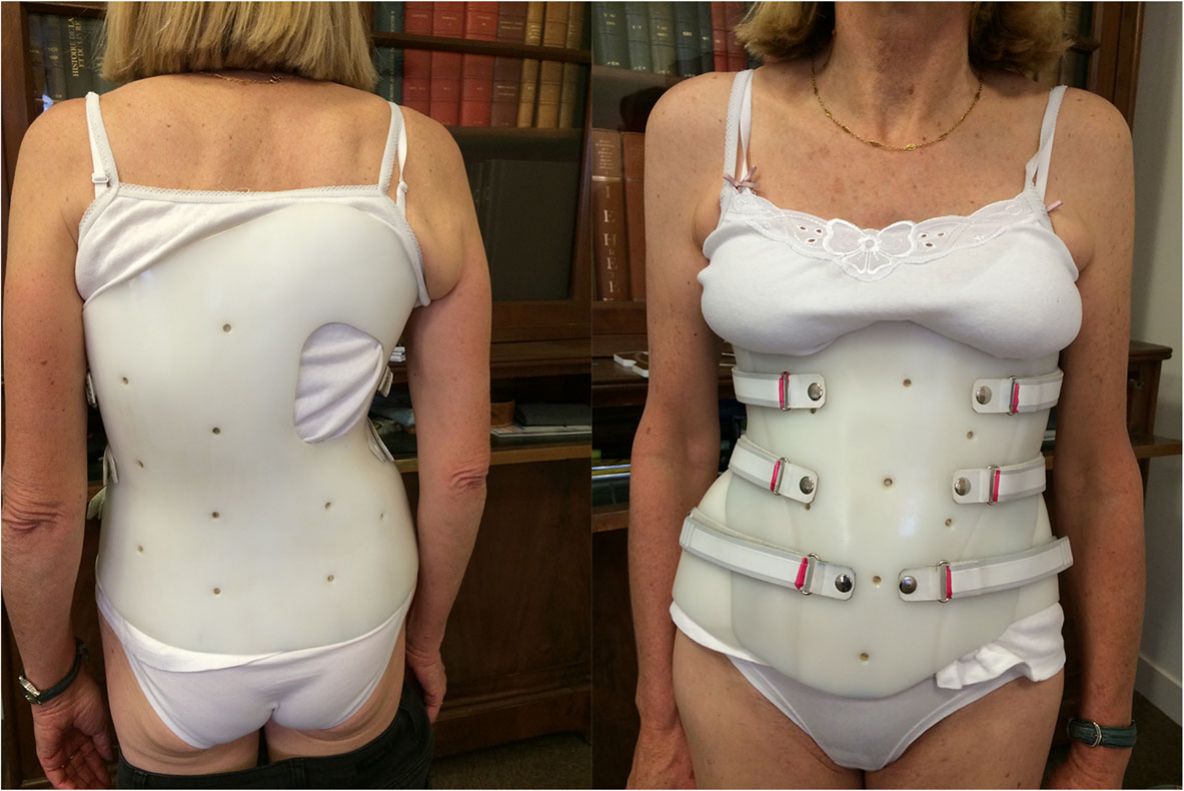
Same patient with brace. The brace is a classical polyethylene bivalve overlapping brace of 3 mm

The Lyon Conservative treatment requires: 1. A plaster cast made in a specific standing frame for 3 weeks. 2. A rigid polyethylene bivalve overlapped brace worn for at least 4 h per day. 3. A specific physiotherapy to prevent muscle atrophy [5]. The plaster cast is an indispensable prerequisite for this treatment. Besides the therapeutic role of muscular-ligamentous adjustment of paravertebral tension, it can also be used as a test. The patient must be pain-free while pursuing normal activities. The rigid brace is usually short, the upper limit being at the thoracic base under the breast. (Figs. 1 and 2) When there is a high thoracic kyphosis, the anterior limit is high at the sternoclavicular level.

## Results

Descriptive parameters are grouped in (Table 2).

**Table 2:** Descriptive parameters. Frequency, mean and standard deviation by gender, age and sex of adult scoliosis

Gender	Female	644
	Male	81
Age	56.80 ± 15.83 (Min 16–Max 91)	725
Cobb angle	35.51 ± 17.18 (Min 10–Max 143)	725

In 14 cases, information on age or Cobb angle was incomplete.

The number of non-adherent patients, ie patients who do not respond to treatment indicated, is 183/739 = 17 % (Table 3).

**Table 3:** Descriptive parameters of drop out group. There is no significant difference regarding age and sex between the 2 groups of drop outs and patients who have completed the proposed treatment

Non-adherent	Age = 58.49 ± 14.74 ns (*p* = 0.201)	Cobb = 36.49 ± 18.20 ns (*p* = 0.637)	138
Non DO	Age = 56.58 ± 16.05 ns (*p* = 0.178)	Cobb = 35.38 ± 17.36 ns (*p* = 0.647)	661

There is no statistically significant difference between the 2 groups of patients.

In adulthood, it is difficult to talk about non-compliance because the wear time of the brace is 4 h a day for six months and then the brace is worn to the patient’s request.

Regroupings according to the aetiology when n > 20 are compared with the group of patients constituting the whole of Statistics and summarized in (Table 4).

**Table 4:** Diagnostic regrouping. The 6 main diagnoses were studied according to the mean and standard deviation of age and Cobb angulation. Each diagnosis is compared with the general statistics which is the control group (*t* test)

All patients	Age 56.97 ± 15.82	Cobb 35.58 ± 17.35
1 - After surgery	53.09 ± 12.91 (*p* = 0.01)*	40.49 ± 15.38 (*p* = 0.01)*
2 - Rotatory Dislocation	59.73 ± 13.52 (*p* = 0.05)	39.08 ± 16.59 (*p* = 0.02)*
3 - Lumbar Instability	46.03 ± 15.49 (*p* = 0.00)*	25.29 ± 12.29 (*p* = 0.00)*
4 - Disabling Pain	48.36 ± 13.73 (*p* = 0.02)*	36.45 ± 25.21 (*p* = 0.78)
6 - Campto-cormia	69.78 ± 12.19 (*p* = 0.00)*	38.09 ± 14.23 (*p* = 0.25)
7 - Thoraco-lumbar kyphosis	60.73 ± 15.51 (*p* = 0.07)	43.34 ± (21.48 (*p* = 0.00)*

*Significant *P* value

The results of patients who completed the treatment will be presented in another publication.

## Discussion

The group after surgery is different from the overall average. Patients are younger and the angle is more important. These results confirm that the decompensation under arthrodesis is faster. The most significant angulation may also explain the quickest decompensation. The brace is not always an alternative to surgery, it can complement and in some cases avoid multiple re-interventions.

The group with rotatory dislocation is the largest and constitutes almost half of the patients. The average age is borderline with statistical significance, the average angulation is significantly higher. The diagnosis is performed on the X-ray with displacement of the spinous processes. Rotatory dislocation is a specific complication of lumbar scoliosis and difficulty of treatment in adulthood justify a conservative treatment during adolescence with short braces.

The group with lumbar instability is the youngest group and the angulation is the lowest which does not justify surgery. The diagnosis is made clinically with pain and mostly a dysfunctional anterior lateral inflexion of the trunk. This instability can be discal or ligamentous in origin. There is no radiological dislocation. Treatment is important because low back pain is the leading cause of disability before age 45.

The group with disabling pain is also younger, but the angulation is not statistically different from the overall average. The diagnosis is difficult without clinical dysfunction and no particular radiological abnormalities. It is the failure of conventional treatments that can justify bracing.

The camptocormia group is the oldest, but the angle was not statistically different. The difficulty in this group, besides age is that camptocormia is most often accompanied by extrapyramidal depression, with less bracing motivation.

For the group with thoracolumbar kyphosis, age is close to the overall average, but angulation significantly greater. The difficulty is the poor tolerance of the sternoclavicular thrust.

The small number of patients with spondylolisthesis confirms the positive evolution of this disease in adulthood. The spinal stenosis often evolves quickly and then the indication is surgical.

All groups are statistically different in terms of age and Cobb angle. The Conservative orthopaedic treatment should be adapted according to these two criteria.

The results of treatment are not the subject of this study.

## Conclusions

The descriptive parameters are used to specify the usual indications of bracing 739 scoliosis. The ratio of women (88 %) is higher than during adolescence. The average age is 57 years and the angulation of 35.5°. The rate of non-adherent patients is 17 %.

The statistical study based on the aetiology enabled to individualize 6 characteristic groups depending on the age and Cobb angle: after surgery, rotatory dislocation, lumbar instability, disabling pain, camptocormia and thoraco-lumbar kyphosis. The differences in age and initial angle are significant. The number of Spinal stenosis and spondylolisthesis is very low in this study.

## Additional file

Additional file 1: Excel spreadsheet: Spreadsheet used for the diagnostic comparison of average according to the age and angulation. (XLSX 80 kb)
